# Feed and production efficiency of young crossbred beef cattle stratified on a terminal total merit index^1^

**DOI:** 10.1093/tas/txaa106

**Published:** 2020-07-01

**Authors:** David N Kelly, Stephen B Conroy, Craig P Murphy, Roy D Sleator, Donagh P Berry

**Affiliations:** 1 Animal and Grassland Research and Innovation Centre, Teagasc, Moorepark, Fermoy, Co. Cork, Ireland; 2 Department of Biological Sciences, Cork Institute of Technology, Bishopstown, Cork, Co. Cork, Ireland; 3 Irish Cattle Breeding Federation, Highfield House, Shinagh, Bandon, Co. Cork, Ireland

**Keywords:** carcass, genetic evaluation, residual feed intake, selection index

## Abstract

Few studies have attempted to quantify the association between a terminal total merit index with phenotypic feed and production efficiency in beef cattle, particularly when feed efficiency is itself explicitly absent as a goal trait in the index. The objective of the present study was to quantify the differences in phenotypic performance for feed intake, feed efficiency, and carcass traits of crossbred bulls, steers, and heifers differing in a terminal total merit index. A validation population of 614 bulls, steers, and heifers that were evaluated for feed intake and efficiency in the same feedlot and subsequently slaughtered at the end of their test period was constructed. The Irish national genetic evaluations for a terminal index of calving performance, docility, feed intake, and carcass traits were undertaken with the phenotypic records of animals present in the validation population masked. The validation population animals were subsequently stratified into four groups, within sex, according to their terminal index value. Mixed models were used to quantify the association between terminal genetic merit and phenotypic performance; whether the associations differed by sex were also investigated. The regression coefficient of phenotypic feed intake, carcass weight, carcass conformation, or carcass fat on its respective estimated breeding values was 0.86 kg dry matter 0.91 kg, 1.01 units, and 1.29 units, respectively, which are close to the expectation of one. On average, cattle in the very high terminal index stratum had a 0.63 kg DM/d lower feed intake, a 25.05 kg heavier carcass, a 1.82 unit better carcass conformation (scale 1 to 15), and a 1.24 unit less carcass fat score (scale 1 to 15), relative to cattle in the very low terminal index stratum. Cattle of superior total genetic merit were also more feed efficient (i.e., had a lower energy conversion ratio, lower residual feed intake, and greater residual gain), had a greater proportion of their live-weight as carcass weight (i.e., better dressing percentage) and were slaughtered at a younger age relative to their inferior total genetic merit counterparts. This study provides validation of an all-encompassing total merit index and demonstrates the benefits of selection on a total merit index for feed and production efficiency, which should impart confidence among stakeholders in the contribution of genetic selection to simultaneous improvements in individual animal performance and efficiency.

## INTRODUCTION

Breeding objectives globally are used to select for improved performance and efficiency (Amer et al., 2001; Berry et al., 2019a). The genetic merit of individual animals for a whole series of traits can be collapsed into a single index value, with the weighting on each trait in the index being a function of its relative importance (Hazel and Lush, 1942). Index weights are usually based on relative economic importance (Amer et al., 2001; Berry et al., 2019a). The direction of genetic change in a trait within the breeding objective should ideally be in the direction of the weighting factor (i.e., negative or positive) but this might not necessarily be the case given the underlying genetic covariances and emphasis on the trait itself as well as on correlated traits. Moreover, genetic change for traits not explicitly included in the breeding objective is also expected to occur if correlated with the overall breeding objective. Furthermore, some traits may be linear combinations of individual traits. One such example is the popular feed efficiency trait residual feed intake (RFI; Byerly, 1941; Koch et al., 1963) which is a linear combination of feed intake, average daily gain (ADG), and metabolic live-weight (MBW), as well as often some measure of body fat (Berry and Crowley, 2013). Van der Werf (2004) demonstrated the mathematical equivalence of including an RFI-type trait as a trait in a breeding goal vs. including the individual component traits. Assuming that all parameters are known, the approach taken is actually at the discretion of the relevant stakeholders (Berry et al., 2015).

The association between genetic merit for individual traits and phenotypic performance in beef cattle has been investigated previously (Crews, 2002; McHugh et al., 2014; Judge et al., 2019) and several studies have demonstrated the favorable effect of selection for improved animal performance using overall genetic merit selection indexes (Connolly et al., 2016; Berry and Ring, 2020). Connolly et al. (2016) for example, using a national database, illustrated that genetically elite cattle (i.e., top 25% for a terminal index) had, on average, heavier and better conformed carcasses, with less fat cover, relative to animals of low genetic merit (i.e., bottom 25% for a terminal index). Berry et al. (2019b) extended this, documenting heavier yields of higher value primal carcass cuts in cattle excelling on the Irish terminal index. Nevertheless, apart from experimental studies with a relatively small number of animals (Clarke et al., 2009), few have attempted to quantify the association between animal total merit index, feed intake, and efficiency in beef cattle.

The objective of the present study was to fill a void in the scientific literature by quantifying the differences in phenotypic performance, especially feed and production efficiency-related traits as well as carcass-related traits, in young crossbred bulls, steers, and heifers differing in total genetic merit for a terminal index. The terminal index investigated was that used in Ireland, which is typical of terminal-type indexes used globally, constituting traits associated with calving performance, feed intake, and carcass merit, as well as some ancillary traits such as docility. Of particular interest in the present study is whether feed efficiency is improving in a selection index that does not explicitly include a feed efficiency trait, but instead includes feed intake alongside output-related traits (i.e., carcass weight corrected to a constant age).

## MATERIALS AND METHODS

The data used in the present study were obtained from an existing database managed by the Irish Cattle Breeding Federation (ICBF). Therefore, it was not necessary to obtain animal care and use committee approval in advance of conducting this study.

### National Genetic Evaluations

The Irish terminal index is an economic-based selection index designed to identify animals excelling genetically in expected profitability of their progeny at slaughter. This index has been implemented and operated by the ICBF in Ireland since 2012. Genetic evaluations in Ireland are multibreed and are undertaken on carcass, live-animal linear scores, feed intake, docility, calving performance, milking ability, and cow fertility traits using the MiX99 software suite (MiX99 Development Team, 2015). As the vast majority of beef cattle in Ireland are crossbred, and a substantial transfer of genetic material takes place between Irish beef and dairy herds (Berry et al., 2006), all genetic evaluations adjust for heterosis and recombination loss, as well as breed differences, through the use of genetic groups in the genetic evaluations. Further details on the national beef genetic evaluations are provided in Evans et al. (2007, 2009).

Estimated breeding values (EBVs) for feed intake [kg dry matter (DM)/d], carcass weight (kg), carcass conformation [scale 1 (poor) to 15 (excellent)], and carcass fat [scale 1 (thin) to 15 (fat)] are estimated in a 29 × 29 multitrait genetic evaluation which also includes six live-weight traits, three animal auction price traits, three composite linear-scored traits, ultrasound muscle depth, three cull cow carcass traits, four primal-cut yield traits, as well as foreign EBVs. Estimated breeding values for docility in growing animals are obtained using a multitrait evaluation that includes the traits of producer-scored weanling docility (scale 1 to 5), weanling docility scored by professional classifiers (scale 1 to 10), and producer-scored cow docility (scale 1 to 5). All docility traits were subjectively assessed, with 1 representing aggressive animals and higher scores representing more docile animals. The EBVs for calving difficulty, mortality, and gestation length are estimated in a 7 × 7 multitrait evaluation that includes direct calving difficulty, birth weight, and other predictor live-weight traits, as well as carcass weight. The current national genetic evaluations use approximately 5.7 million calving records, 8.6 million carcass records, 1.9 million weanling docility records, and 6,286 feed intake records. Feed intake records used in the national genetic evaluation originate from the national bull performance test station (pre-2012) which was replaced by the national progeny performance test station (post-2011). The protocols, diets, and data editing procedures are described, in detail, by both Crowley et al. (2010) and Kelly et al. (2019) but are also outlined briefly here.

### Phenotypic Data Used in the Present Study

Feed intake, live-weight, carcass, and ultrasound data were available for animals that were on test for feed intake at the National Bull Performance Test Centre (1983 to 2011) and ICBF Gene Ireland Progeny Test Centre (2012 to present); no feed intake, live-weight, or ultrasound data were available for the transitionary period of October 2011 to July 2012.

Prior to 2012, bulls entered the test station in, on average, three different strata annually, hereafter referred to as batches. There were 2 to 5 bulls per pen, assigned based on breed and live-weight, and all 40 pens were equipped with a Calan Broadbent gate system (American Calan, Northwood, NH) for recording individual bull feed intake. Bulls were initially fed 4.5 to 6.0 kg of concentrates daily, an allowance which was increased daily by 10% of the previous day’s allowance. The test started once ad libitum levels of feed intake were reached and the test period ranged from 83 to 225 d long. A daily allowance of 1.5 kg fresh weight of hay per bull was added to the Calan Broadbent feeder throughout the test period and access to clean fresh water was also provided ad libitum. Animals were weighed fortnightly between 1992 and 1995, every 21 d between 1995 and 2005, fortnightly between 2005 and 2008 and every 21 d between 2008 and 2011. All hay was assumed to have a DM of 85% and a metabolizable energy concentration of 8.6 MJ/kg DM. The concentrates offered to bulls between September 1992 and September 2002 was assumed to have a DM of 87.5% and a metabolizable energy concentration of 12.1 MJ/kg DM, whereas the concentrates offered to bulls between October 2002 and September 2011 was assumed to have a DM of 86% and a metabolizable energy concentration of 14.5 MJ/kg DM. Where feed energy composition was available, daily metabolizable energy intake (MEI) for each bull was calculated as the sum of daily hay dry matter intake (DMI) multiplied by hay metabolizable energy concentration and daily concentrate DMI multiplied by concentrate metabolizable energy concentration. Further details of the feeding and management of animals, while in the acclimatization period and while on test, are described in detail by Crowley et al. (2010).

The test center changed function in 2012 from a performance test center to a progeny test center, accommodating sexes other than bulls. Young bulls, steers, and heifers were purchased by the ICBF from Irish commercial cattle producers from August 2012 onwards. The animals entered the progeny test station in batches, where they were evaluated for feed intake and growth. All animals within each batch started their test together and all animals within a batch were slaughtered within a week of each other at the end of their test period. Each batch was composed of one sex and was grouped by birth-date where the maximum range in age was 2 mo. On arrival at the test center, all cattle were placed in pens, based on live-weight and breed, and subsequently underwent an acclimatization period of between 21 and 30 d, to adapt to the feeding system and environment. The actual test period ranged from 77 to 90 d. There were 4 to 6 animals per pen, across a total of 40 pens; 30 pens were equipped with 2 automatic feed stations (RIC Feed-Weigh Trough, Hokofarm Group BV, Marknesse, The Netherlands) and a further 10 pens were equipped with a Calan Broadbent gate system (American Calan, Northwood, NH). Only young bulls were fed through the Calan Broadbent gate system while all steers, heifers, and some bulls were fed through the automatic feed stations. Access to clean, fresh water was provided ad libitum for every pen in the test center, with one water trough shared between two adjacent pens.

In the test station, all animals were weighed, on average, every 7 d between August 2012 and August 2013, every 21 d between September 2013 and December 2017, and every 7 d in 2018 and 2019. All hay fed to young bulls, steers, and heifers was assumed to have a DM of 86% and a metabolizable energy concentration of 8.6 MJ/kg DM. The concentrates offered to bulls between August 2012 and July 2019 was assumed to have DM of 86% and a metabolizable energy concentration of 14.1 MJ/kg DM. Daily MEI for each bull was calculated as the sum of daily hay DMI multiplied by hay metabolizable energy concentration and daily concentrate DMI multiplied by concentrate metabolizable energy concentration. The total mixed ration fed to all steers and heifers was assumed to have a DM of 51% and a metabolizable energy concentration of 12.1 MJ/kg DM. For steers and heifers, the daily MEI per animal was defined as daily DMI multiplied by the energy concentration of the total mixed ration. All hay DM values were provided by the hay supplier and the appropriate hay energy values were derived from feed tables (Sauvant et al., 2004); concentrate energy and DM values were obtained from the feed manufacturer. Further details of the feeding and management of animals while in the acclimatization period and while on test, as well as the operation of the automatic feed stations, are described in detail by Kelly et al. (2019).

### Validation Dataset

For the purposes of constructing a validation population, only animals that were on test for feed intake at the ICBF Progeny Test Centre and slaughtered at the end of their test between the calendar years of 2017 to 2019, inclusive, were considered. Additionally, only animals with known parents were retained and data from 174 animals that were direct progeny of a non-beef dam (i.e., Friesian, Friesian cross, Jersey, Jersey cross, Montbeliarde, Montbeliarde cross, Norwegian Red, Norwegian Red cross, Rotbunte, and Rotbunte cross) were removed from all analyses, as they would have experienced a very different early-life production system which would then be confounded with total merit. All animals had to have at least three live-weight records taken during the test period; the most recent live-weight before the test period was retained if it was recorded within 7 d of the start of the test period. Data from 11 animals were removed due to abnormal growth rates, where the *r*-squared of a linear regression through their live-weight records was <0.90, as discussed by Kelly et al. (2019). Data from eight animals identified as sick from a combination of their growth and live-weight records were also removed from all analyses.

Following all edits, feed intake, live-weight, and carcass-related records were available on 614 animals, 234 of which were young bulls, 129 were heifers, and 251 were steers. In order to maximize the number of feed intake records used in the genetic evaluation, the national genetic evaluations for carcass traits and feed intake were rerun three times. In each instance, the phenotypes of either the 234 young bulls, the 129 heifers, or the 251 steers in the validation dataset were masked such that their EBVs were calculated from relatives with phenotypes. The national evaluations for calving traits and docility were executed with the phenotypes of animals in the validation dataset also masked in the evaluation. Approximate reliabilities were generated in MiX99 which used the method described by Tier and Meyer (2004), as per the Irish national genetic evaluations. A terminal index for each animal was then constructed as the sum of each trait’s EBV multiplied by the respective economic weight generated from the Grange Beef Model (Crosson et al., 2006) and used in the construction of the current Irish terminal index:

$$\begin{array}{l} \begin{array}{lll} Terminal\;index & = & \left(-\text{\textbackslash{}euro}4.65\times calving\;difficulty\;EBV\right)\\ \; & \; & +(-\text{\textbackslash{}euro}2.25\times gestation\;length\;EBV)\\ \; & \; & +(-\text{\textbackslash{}euro}5.34\times mortality\;EBV)\\ \; & \; & +(\text{\textbackslash{}euro}17.02\times docility\;EBV)\\ \; & \; & +(-\text{\textbackslash{}euro}38.63\times feed\;intake\;EBV)\\ \; & \; & +(\text{\textbackslash{}euro}3.14\times carcass\;weight\;EBV)\\ \; & \; & +(\text{\textbackslash{}euro}14.77\times carcass\;conformation\;EBV)\\ \; & \; & +(-\text{\textbackslash{}euro}7.86\times carcass\;fat\;EBV). \end{array} \end{array}$$

The economic weights and relative emphasis for each trait in the terminal index are summarized in Table 1. It should be noted that the actual deployed terminal index in Ireland is based on predicted transmitting ability values but, for the present study, EBVs were used since the animals themselves (i.e., not their progeny) are the experimental unit for validation. The validation population animals were stratified, within sex, into four terminal index strata of equal size based on their calculated total merit index as: 1) very high terminal index, 2) high terminal index, 3) low terminal index, and 4) very low terminal index. A new variable was created per animal, which was a covariate expressed relative to a certain terminal index value within each stratum, so that the difference in terminal index between all adjacent strata was equivalent. This covariate was used in the statistical models with the objective of enabling the testing for linearity of associations across all strata.

**Table 1.** The economic weights and relative emphasis of the traits included in the terminal index

Trait	Economic weight, €/trait unit	Relative emphasis, %
Calving difficulty, %^1^	−4.65	18
Gestation length, d	−2.25	4
Mortality, %^2^	−5.34	3
Docility, scale 1–5^3^	17.02	2
Daily feed intake, kg DM/d	−38.63	16
Carcass weight, kg	3.14	41
Carcass conformation, scale 1–15^4^	14.77	11
Carcass fat, scale 1–15^4^	−7.86	5

^1^Percentage of progeny records that are 3 or 4 on a 1–4 scale; a score of 1 represents a normal calving and a score of 4 represents veterinary assistance at calving.

^2^Percentage of calves stillborn or dead within 5 d of birth.

^3^A score of 1 represents aggressive animals and a score of 5 represents docile animals.

^4^A score of 1 represents poor conformation or a lean carcass, and a score of 15 represents a well conformed or fat carcass.

### Trait Definitions

Carcass weight (kg) was measured, on average, 1 h postslaughter. Video image analysis in a mechanical grading system was used to determine the carcass conformation and carcass fat class of each animal which were both defined using the EUROP carcass classification system (Pabiou et al., 2011). Carcass conformation and carcass fat were both represented on a scale from 1 to 15 (Englishby et al., 2016). For both scales, a score of 1 denotes poor conformation and a low level of fat cover, while a score of 15 denotes excellent conformation and a high level of fat cover. Ultrasound measurements of fat depth, eye muscle depth, and intramuscular fat were recorded on the live animals as described by Kelly et al. (2019). In the present study, only 422 animals from the validation population had ultrasound records, and just the last record of each ultrasound measurement preslaughter was retained for each animal. As defined by Coyne et al. (2019), dressing difference (kg) was calculated as the animal’s final live-weight preslaughter, minus its carcass weight. Dressing percentage (%) was defined as the carcass weight divided by the animal’s final live-weight preslaughter. Five days was the longest time period between the recording of preslaughter live-weight and carcass weight in the present study.

Average daily gain was calculated, per animal, as the linear regression coefficient from a simple linear regression of live-weight records on days on test (Kelly et al., 2019). Midtest metabolic live-weight (i.e., live-weight^0.75^) was represented as the predicted MBW 35 d before the end of the test, derived from the intercept and linear regression coefficient of MBW measures on days on test. Energy conversion ratio (ECR) was defined as MEI divided by ADG; Kleiber ratio was calculated as ADG divided by MBW and relative growth rate (RGR) was calculated as:

$$\begin{array}{ll} & RGR=100\times \\ & \left(\frac{\left[log_{e}(end\;test\;liveweight)-log_{e}(start\;test\;liveweight)\right]}{days\;on\;test}\right). \end{array}$$

Residual feed intake is defined as the difference between each animal’s actual energy intake and their predicted energy intake (Koch et al., 1963; Arthur et al., 2001). In the present study, residual energy intake (REI) was calculated using the same principle but where feed intake was substituted by energy intake. The traditional definition of REI was calculated as the residuals from a multiple linear regression of MEI on both MBW and ADG:

$$\begin{array}{l} REI=MEI-(\beta_{0}+\beta_{1}MBW+\beta_{2}ADG+batch) \end{array}$$

where *β*_0_ represents the intercept and *β*_1_ and *β*_2_ represent the respective partial regression coefficients of MEI on MBW and ADG. Where ultrasound records were available, a separate trait of REI adjusted for ultrasound fat depth (REI_U_) was calculated as already described for REI except ultrasound fat depth was itself included as a covariate as well as in a two-way interaction with both ADG and MBW (Savietto et al., 2014). Residual gain (RG) is defined as the difference between an animal’s actual growth rate and predicted growth rate (Koch et al., 1963; Crowley et al., 2010) and was calculated as the residuals from a multiple linear regression of ADG on both MBW and MEI:

$$\begin{array}{l} RG=ADG-(\beta_{0}+\beta_{1}MBW+\beta_{2}MEI+batch) \end{array}$$

where *β*_0_ represents the intercept and *β*_1_ and *β*_2_ represent the respective partial regression coefficients of ADG on MBW and MEI. Where ultrasound records were available, a separate trait of RG adjusted for ultrasound fat depth (RG_U_) was calculated as already described for RG except that ultrasound fat depth was itself also included as a covariate and in a two-way interaction with both MEI and MBW. Residual intake and gain (RIG) was calculated as RG − REI, each standardized to a variance of 1 (Berry and Crowley, 2012). Additionally, RIG adjusted for ultrasound fat depth (RIG_U_) was calculated as RG_U_ − REI_U_, each standardized to a variance of 1.

A general heterosis and recombination loss coefficient were calculated for each animal as:

$$\begin{array}{l} 1-\sum_{i=1}^{n}sire_{i}\times dam_{i}\; \end{array}$$

and

$$\begin{array}{l} 1-\;\sum_{i=1}^{n}\frac{\left({sire}_{i}^{2}\times {dam}_{i}^{2}\right)}{2} \end{array}$$

where sire_*i*_ and dam_*i*_ are the proportion of breed *i* in the sire and dam, respectively (Van Raden and Sanders, 2003). Heterosis was divided into 12 classes (0%, >0% and ≤10%, >10% and ≤20%, …, >90% and <100%, and 100%), and recombination loss was divided into seven classes (0%, >0% and ≤10%, >10% and ≤20%, >20% and ≤30%, >30% and ≤40%, >40% and ≤50%, and >50%).

### Statistical Analyses

The associations between terminal index stratum and each phenotypic performance trait were determined using linear mixed models with PROC MIXED in SAS (SAS Institute Inc., Cary, NC). Fixed effects included in all models were terminal index stratum (very high, high, low, and very low), dam parity (1, 2, 3, 4, and ≥5), animal sex (bull, steer, or heifer), heterosis class, recombination loss class, and month of age at slaughter (except when age at slaughter was the dependent variable). Batch (i.e., contemporary group) was included as a random effect in all analyses, apart from when any of the residual-related efficiency traits were the dependent variable since batch had already been considered in their derivation. A two-way interaction between terminal index stratum and animal sex was also tested to evaluate if the association between performance and terminal index differed by animal sex. In a subsequent analysis, carcass weight was also included as a covariate and in a two-way interaction with sex when age at slaughter was the dependent variable. When terminal index was included in the model as a class variable, the previously defined covariate was also fitted which reflected the difference between each animal’s terminal index value relative to a value within each stratum such that the difference in terminal index value between each adjacent stratum was equivalent. In a separate series of analyses, the independent variable of terminal index stratum was replaced with the continuous variable for either terminal index, feed intake EBV, carcass weight EBV, carcass conformation EBV, or carcass fat EBV.

## RESULTS

The average age at slaughter in the current study was 461 d (SD = 26.28 d) for bulls, 537 d (SD = 56.11 d) for heifers, and 572 d (SD = 45.24 d) for steers. The average carcass weight was 388.4 kg (SD = 49.20 kg), 325.1 kg (SD = 37.50 kg), and 359.9 kg (SD = 37.91 kg) for bulls, heifers, and steers, respectively. The mean reliability estimate for the EBVs of the validation animals for feed intake, carcass weight, carcass conformation, and carcass fat was 0.19 (SD = 0.03), 0.30 (SD = 0.02), 0.29 (SD = 0.03), and 0.28 (SD = 0.03), respectively.

### Impact of Terminal Index Strata

The difference in mean terminal index value between adjacent terminal index strata was €43.72, which is marginally greater than half the SD of the terminal index; the SD of the current Irish terminal index, on an EBV scale, is €81. The associations between all of the performance, efficiency, carcass, and ultrasound traits with terminal index as a class variable did not differ (*P* > 0.05) by sex in the present study; hence, only results across all sexes are discussed further. Nonetheless, the least squares means calculated within each sex (bull, steer, and heifer) for the performance, efficiency, carcass, and ultrasound traits are presented in Supplementary Tables S1 to S3. Furthermore, because all animals were treated the same, one could infer that the associations detected with genetic merit are more than likely causal.

Least squares means for performance, efficiency, carcass, and ultrasound traits for animals stratified by terminal index across sexes are summarized in Table 2. Across all of the terminal index strata, cattle did not differ (*P* > 0.05) in ADG, MBW, or final live-weight preslaughter. Cattle in the top 25% for the terminal index, however, consumed 0.63 kg DM/d (SED = 0.17 kg DM/d) less, and correspondingly 7.97 MJ/d (SED = 2.12 MJ/d) less than cattle in the bottom 25%. Furthermore, relative to cattle in the very low genetic merit stratum, cattle in the very high genetic merit stratum were slaughtered 7.17 d (SED = 2.81 d) younger. The difference in age at slaughter between extreme terminal index strata increased to 10.23 d (SED = 2.85 d) when carcass weight was included as a covariate in the model. Both age at slaughter and age at slaughter adjusted to a common carcass weight increased approximately linearly with increasing terminal index value.

**Table 2.** Least squares phenotypic means^1^ and pooled standard errors of the difference between least squares means (SED) for performance, efficiency, carcass, and ultrasound traits for very low, low, high, and very high terminal index animals

Trait^2^	Very low	Low	High	Very high	SED
Performance					
Average daily gain, kg/d	1.57	1.59	1.57	1.63	0.033
Dry matter intake, kg/d	12.59^a^	12.44^ab^	12.03^bc^	11.96^c^	0.169
Metabolizable energy intake, MJ/d	158.30^a^	156.56^ab^	151.36^bc^	150.33^c^	2.113
Metabolic live-weight, kg^0.75^	120.0	121.9	119.9	121.0	1.033
Preslaughter live-weight, kg	651.5	663.7	649.9	659.5	7.274
Age at slaughter^3^, d	529^a^	527^a^	525^a^	522^b^	2.805
Adjusted age at slaughter^4^, d	531^a^	527^ab^	525^bc^	521^c^	2.787
Efficiency					
Energy conversion ratio	106.03^a^	103.92^a^	101.47^ab^	96.32^b^	1.965
Relative growth rate	0.290	0.289	0.287	0.299	0.006
Kleiber ratio	0.0131	0.0131	0.0130	0.0136	0.0003
Residual energy intake, MJ/d	3.42^a^	0.03^b^	−2.70^b^	−5.64^c^	1.477
REI_U_, MJ/d	1.27^a^	0.06^ab^	−1.43^ab^	−4.64^b^	1.795
Residual gain, kg/d	−0.02^a^	−0.01^a^	0.00^a^	0.08^b^	0.028
RG_U_, kg/d	−0.02^a^	−0.01^a^	−0.01^a^	0.05^b^	0.031
Residual intake and gain	−0.41^a^	−0.03^ab^	0.25^b^	0.85^c^	0.198
RIG_U_	−0.21^a^	−0.06^a^	0.07^a^	0.69^b^	0.259
Carcass					
Carcass weight, kg	357.21^a^	369.80^b^	370.58^b^	382.23^c^	4.339
Carcass conformation, scale 1–15	9.33^a^	10.05^b^	10.63^c^	11.16^d^	0.146
Carcass fat, scale 1–15	7.83^a^	7.44^b^	6.96^bc^	6.59^c^	0.154
Dressing difference, kg	293.89^a^	293.44^a^	278.78^b^	276.92^c^	3.625
Dressing percentage, %	54.90^a^	55.75^b^	57.14^c^	58.12^d^	0.247
Ultrasound					
Ultrasound fat depth, mm	5.45^a^	4.92^b^	4.23^c^	3.87^c^	0.189
Ultrasound muscle depth, mm	75.22^a^	77.07^ab^	79.76^bc^	80.90^c^	0.939
Intramuscular fat, %	6.04^a^	5.84^ab^	5.43^bc^	5.15^c^	0.174

^a–d^Least squares means within a row with different subscripts differ (*P* < 0.05).

^1^Referrent animal was a purebred steer from a third-parity dam slaughtered at 20 mo of age.

^2^REI_U_ = residual energy intake adjusted for ultrasound fat depth; RG_U_ = residual gain adjusted for ultrasound fat depth; RIG_U_ = residual intake and gain adjusted for ultrasound fat depth.

^3^Referent animal was a purebred steer from a third-parity dam.

^4^Referent animal was a purebred steer from a third-parity dam slaughtered at a carcass weight of 360 kg.

There were no differences in RGR or Kleiber ratio between the different terminal index strata, but for all other feed efficiency traits, the cattle in the very high genetic merit stratum were more feed efficient than cattle in the very low genetic merit stratum; in fact, the least squares mean for these efficiency traits increased linearly per incremental improvement in terminal index stratum. Relative to cattle in the very low terminal index stratum, cattle in the very high stratum had a 9.71 unit (SED = 1.97 unit) lower ECR, a 9.06 MJ/d (SED = 1.47 MJ/d) lower REI, and 0.10 kg/d (SED = 0.027 kg/d) greater RG.

Cattle in the very high terminal index stratum had heavier, leaner, and more conformed carcasses relative to cattle in the very low terminal index stratum. Cattle in the very high terminal index stratum had, on average, a 25.02 kg (SED = 4.35 kg) heavier carcass weight, a 16.97 kg (SED = 3.63 kg) lighter dressing difference, and subsequently a 3.22% (SED = 0.25%) greater dressing percentage, relative to cattle in the very low terminal index stratum. The difference in least squares means between adjacent strata on terminal index was approximately the same going from very low to very high index. Higher index cattle had a greater ultrasound muscle depth (*P* < 0.001) and both a lower ultrasound fat depth (*P* < 0.001) and a lower intramuscular fat percentage (*P* < 0.001) than cattle of lower total genetic merit.

### Effect of Terminal Total Genetic Merit as a Continuous Variable

The phenotypic change in the performance, efficiency, ultrasound, and carcass traits with each unit change in terminal index value is summarized in Table 3. The phenotypic change in ADG, MBW, preslaughter live-weight, Kleiber ratio, and RGR for each unit change in terminal index value was not different (*P* > 0.05) from zero, consistent with what was observed when genetic merit was treated as a class effect. A €10 greater terminal index was associated with a 0.69 MJ/d (SE = 0.15 MJ/d) lower MEI and a 0.055 kg DM/d (SE = 0.012 kg DM/d) lower DMI. Every €10 increase in terminal index value contributed to a 0.69 unit (SE = 0.14 unit) lower ECR, a 0.70 MJ/d (SE = 0.10 MJ/d) lower REI, and a 0.092 unit (SE = 0.014 unit) greater RIG. The phenotypic change in each of REI, RG, RIG, and RIG_U_ for each unit change in terminal index was in the same direction for all sexes but did differ (*P* < 0.05) between bulls and steers with a stronger effect in bulls. The absolute value of the phenotypic change in age at slaughter with every unit increase in terminal index increased from 0.058 d (SE = 0.020 d) to 0.079 d (SE = 0.020 d) when carcass weight was included as a covariate in the mixed model. As terminal index value increased, phenotypic carcass weight increased (*P* < 0.001) concomitant with a reduction (*P* < 0.001) in dressing difference. Both carcass fat and ultrasound fat depth reduced (*P* < 0.001) with increasing terminal index value, although the change in ultrasound fat depth differed (*P* = 0.011) between bulls and steers; each unit increase in terminal index was associated with a 0.0092 mm (SE = 0.002 mm) reduction and 0.016 mm (SE = 0.002 mm) reduction in ultrasound fat depth for bulls and steers, respectively.

**Table 3.** The phenotypic change (SE in parentheses) in the performance, efficiency, carcass, and ultrasound traits for a one unit change in terminal index for all animals, bulls, heifers, and steers

Trait^1^	All animals	Bulls	Heifers	Steers
Performance				
Average daily gain, kg/d	0.00027 (0.00023)	0.00023 (0.00038)	0.00064 (0.00047)	−0.00006 (0.00033)
Dry matter intake, kg/d	−0.0055 (0.0012)	−0.0064 (0.0019)	−0.0062 (0.0024)	−0.0038 (0.0017)
Metabolizable energy intake, MJ/d	−0.069 (0.015)	−0.089 (0.024)	−0.075 (0.030)	−0.044 (0.021)
Metabolic live-weight, kg^0.75^	0.019 (0.051)	0.005 (0.012)	0.002 (0.015)	−0.0015 (0.010)
Preslaughter live-weight, kg	0.0020 (0.0073)	0.042 (0.083)	0.039 (0.110)	−0.024 (0.072)
Age at slaughter, d	−0.058 (0.020)	−0.024 (0.032)	−0.085 (0.04)	−0.066 (0.027)
Adjusted age at slaughter, d	−0.079 (0.020)	−0.044 (0.032)	−0.11 (0.041)	−0.087 (0.027)
Efficiency				
Energy conversion ratio	−0.069 (0.014)	−0.051^ab^ (0.022)	−0.119^a^ (0.028)	−0.038^b^ (0.019)
Relative growth rate	0.000039 (0.000044)	0.000018 (0.000072)	0.000126 (0.000091)	−0.00003 (0.000062)
Kleiber ratio	0.0000018 (0.0000018)	0.0000009 (0.0000030)	0.0000057 (0.0000038)	−0.0000011 (0.0000026)
Residual energy intake, MJ/d	−0.070 (0.010)	−0.096^a^ (0.017)	−0.076^ab^ (0.021)	−0.036^b^ (0.014)
REI_U_, MJ/d	−0.048 (0.012)	−0.061 (0.019)	−0.066 (0.026)	−0.016 (0.019)
Residual gain, kg/d	0.00066 (0.00019)	0.0011^a^ (0.00032)	0.0007^ab^ (0.0004)	0.0001^b^ (0.00027)
RG_U_, kg/d	0.00044 (0.00022)	0.00079 (0.00033)	0.00065 (0.00045)	−0.00012 (0.00033)
Residual intake and gain	0.0092 (0.0014)	0.013^a^ (0.0023)	0.010^ab^ (0.0028)	0.004^b^ (0.0019)
RIG_U_	0.0068 (0.0018)	0.0097^a^ (0.0027)	0.0096^ab^ (0.0037)	0.0011^b^ (0.0027)
Carcass				
Carcass weight, kg	0.17 (0.031)	0.20 (0.050)	0.17 (0.063)	0.15 (0.043)
Carcass conformation, scale 1–15	0.013 (0.0010)	0.013 (0.0017)	0.014 (0.0021)	0.014 (0.0014)
Carcass fat, scale 1–15	−0.0097 (0.0011)	−0.011 (0.0017)	−0.008 (0.0022)	−0.01 (0.0015)
Dressing difference, kg	−0.15 (0.026)	−0.16 (0.042)	−0.13 (0.052)	−0.17 (0.036)
Dressing percentage, %	0.025 (0.0017)	0.026 (0.0028)	0.025 (0.0035)	0.025 (0.0024)
Ultrasound				
Ultrasound fat depth, mm	−0.012 (0.0013)	−0.0092^a^ (0.0020)	−0.012^ab^ (0.0026)	−0.016^b^ (0.0020)
Ultrasound muscle depth, mm	0.046 (0.0065)	0.058 (0.0099)	0.045 (0.013)	0.035 (0.010)
Intramuscular fat, %	−0.0072 (0.0012)	−0.0076 (0.0018)	−0.0055 (0.0025)	−0.0083 (0.0019)

^a–b^Regression coefficients within a row with difference superscripts differ (*P* < 0.05).

^1^REI_U_ = residual energy intake adjusted for ultrasound fat depth; RG_U_ = residual gain adjusted for ultrasound fat depth; RIG_U_ = residual intake and gain adjusted for ultrasound fat depth.

### Validation of Feed Intake and Carcass-Related EBVs

The phenotypic change in either DM intake, carcass weight, carcass conformation, or carcass fat per unit change in its respective EBV did not differ by animal sex (*P* > 0.05). A 1.0 kg DM/d increase in feed intake EBV was associated with a 0.86 kg DM/d (SE = 0.11 kg DM/d) and an 11.01 MJ/d (SE = 1.41 MJ/d) increase in phenotypic DM intake and MEI, respectively. A one unit increase in carcass weight EBV was associated with, on average, a 0.91 kg (SE = 0.11 kg) increase in phenotypic carcass weight. Each one unit increase in carcass conformation EBV value was associated with a 1.01 unit (SE = 0.061 unit) increase in phenotypic carcass conformation and similarly, a one unit increase in carcass fat EBV was associated with a 1.29 unit (SE = 0.095 unit) increase in phenotypic carcass fat.

## DISCUSSION

Total merit indexes in cattle, especially beef indexes, are not often validated using phenotypic data (Berry and Ring, 2020). Furthermore, the impact of rank for total merit index on phenotypic feed efficiency has not been previously quantified in large cohorts of beef cattle. A plethora of studies have estimated genetic parameters for efficiency traits, such as feed conversion ratio, RFI, and RG, across multiple cattle populations (see Berry and Crowley, 2013 for review), all of which suggest that interanimal genetic differences do exist. Genetic covariances between performance and efficiency traits also exist in cattle (for review see Berry and Crowley, 2013). While the effect of terminal index on both performance and efficiency estimated in the present study could, theoretically, have been deduced from selection index theory, these calculations would need to assume that 1) (co)variance components are known without error, 2) genetic differences translate precisely into phenotypic differences, and 3) the relationships among traits that constitute the index are linear as dictated by the correlations used in the calculations.

Much of the narrative on breeding for feed efficiency revolves around explicit direct selection for a feed efficiency trait such as RFI (Van der Werf, 2004). Nonetheless, Van der Werf (2004) mathematically demonstrated the equivalence of including either RFI or its component traits (i.e., feed intake, growth rate, and MBW) in a selection index, assuming no fixed effects were in the model. The results from the present study confirm this assertion, in that the terminal index used in Ireland has enabled the breeding of more feed efficient (i.e., lower RFI and lower REI) cattle, even when RFI and/or REI has not been explicitly included in the index itself. As RFI may be a relatively difficult concept of feed efficiency to understand by the end-user, including feed intake rather than RFI in a selection index may be considered a more intuitive strategy to implement in a terminal breeding objective (Berry et al., 2015).

Since the unit of genetic merit in the present study was EBV, the close to unity regression coefficient of phenotypic performance on its respective EBV across all sexes was desired and expected, even considering that the EBV reliability estimates were relatively low. Such a relationship between genetic merit and the respective phenotypic performance has been previously verified in cattle by Connolly et al. (2016), Crews (2002) and McHugh et al. (2014). The genetic standard deviation of feed intake in the national genetic evaluation is 1.29 kg DM/d; the expected mean difference between the top and bottom 10% of a normal distribution is 3.51 standard deviation units. Therefore, the expected mean phenotypic difference in EBV for feed intake between extreme deciles is 3.89 kg DM/d (i.e., 1.29 kg DM/d × 3.51 standard deviation units × 0.86 regression coefficient). Such a difference represents 31.7% of the mean feed intake of 12.28 kg DM/d in the present study.

### Benefits of Improved Feed and Production Efficiency Within a Terminal Total Merit Index

Results from the present study clearly demonstrate that the terminal index used in Ireland is enabling the selection of more feed efficient cattle that yield heavier carcasses of better quality; this terminal index is similar to other terminal indexes used globally (Amer et al., 1998; Ochsner et al., 2017). The results from the present study regarding the relationship between terminal index genetic merit and feed efficiency also corroborate those of Clarke et al. (2009), based on a relatively small controlled experiment study of 107 animals divergent for a previous Irish terminal index. Although Clarke et al. (2009) reported that there was no difference in DM intake between progeny of high and low genetic merit sires, RFI was reported to improve with increasing sire genetic merit for their terminal index comprised of weaning weight, DM intake, carcass weight, carcass conformation, and carcass fat. In the terminal total merit index described in the present study, there is a dual benefit for feed and production efficiency; not only are beef cattle of higher total genetic merit more efficient per day, they are also more efficient across the entire finishing period in that they are slaughtered at a younger age, even at a common carcass weight. Cattle in the very high genetic merit strata were 7.47 d (SED = 2.82 d) younger at the start of the test period than cattle in the very low genetic merit strata and, as there was no association between initial test weight and terminal genetic merit in the present study, this suggests that, assuming the same birth weight, the higher terminal index cattle had a better early life or pretest growth rate; such growth rate traits were not analyzed in the present study as no data on birth weight were available. The dual benefit of reduced feed intake per day and reduced number of days of feeding in the high genetic merit cattle also has implications for improved environmental sustainability (Berry et al., 2015).

Donoghue et al. (2016) demonstrated a positive genetic correlation between feed intake and daily methane production in growing beef cattle. Thus, as the higher genetic merit cattle in the current study ate less per day, they are also expected to have a lower daily methane output, relative to their lower genetic merit contemporaries. Furthermore, while the genetically elite cattle are expected to emit less methane per day, because they are slaughtered at a younger age, they would also be producing methane (and other compounds which affect the environment or water quality) for fewer days. Much like the fact that improvements in feed efficiency (i.e., lower REI, lower ECR, and greater RIG) are being achieved in beef cattle in Ireland without the explicit inclusion of a feed efficiency trait in the terminal index, a lower environmental footprint of genetically elite animals is also likely, even without the direct inclusion of an environmental trait in the breeding objective. Undoubtedly, faster genetic gain should be possible by directly including an appropriate trait, such as daily methane output, explicitly reflecting environmental footprint.

### Benefits of Improving Carcass Yield and Quality in a Terminal Total Merit Index

The superior carcass metrics of the animals excelling in the terminal index in the present study have also previously been demonstrated by Connolly et al. (2016). Using a national database, Connolly et al. (2016) reported that cattle in the very high genetic merit stratum for a terminal index had a 37.1 kg heavier carcass, a 1.98 units superior carcass conformation (scale 1 to 15) and a 1.33 units less carcass fat (scale 1 to 15), relative to cattle in the very low genetic merit stratum. The smaller differences between the very high and very low genetic merit strata reported in the present study, for the same index and the same traits, are likely due to the fact that the difference in terminal index value between adjacent terminal index strata in Connolly et al. (2016) was approximately 17% greater than that in the present study (€51.30 vs. €43.72). Although Connolly et al. (2016) did include both beef and dairy origin animals in their study, which would have contributed to the larger phenotypic differences between terminal index strata, terminal index strata in their study were balanced for animal origin to overcome confounding between terminal index strata and whether the animal was born into a beef or dairy herd. Furthermore, Connolly et al. (2016) did observe a 0.017 d (SE = 0.007 d) older age at slaughter in beef origin cattle with each increase in terminal index value, which was in contrast to the 0.058 d younger age at slaughter for each unit increase in terminal index in the current study. This discrepancy may be due to the fact that both dairy and beef animals were together included in the analyses by Connolly et al. (2016).

Where cattle are destined for slaughter, not only are heavier and more conformed carcasses generally desired, but a greater dressing percentage is also preferred as it implies a larger proportion of the animal’s live-weight materializes as saleable carcass yield (Coyne et al., 2019). The higher terminal index cattle in the present study had a greater dressing percentage which was achieved through the generation of not only a heavier carcass, but also a lighter dressing difference, while pre-slaughter live-weight did not differ between terminal index strata. The dressing difference is of little actual value to the producer (Coyne et al., 2019) and, as it is a component of the live-weight of the animal, it also has an associated environmental cost since it reflects a part of the maintenance value of an animal.

Judge et al. (2019) demonstrated that carcass conformation in cattle is strongly genetically correlated to the weight of primal carcass cuts (e.g., striploin, fillet, and topside), even when corrected to a common carcass weight. Therefore, as the higher terminal index cattle in the present study had better carcass conformation, they were expected to yield a greater quantity of higher value primal carcass cuts than their lower terminal index counterparts. In fact, Connolly et al. (2019) reported that animals in the top 25% for a terminal index had a greater predicted yield of higher value carcass cuts as well as more total meat yield, relative to animals in the bottom 25%; cut yields were predicted from video image analysis (Pabiou et al., 2011). Similarly, Berry et al. (2019b), using actual primal-cut weight data, reported that the weight of primal cuts increased almost linearly with increasing genetic merit for a total merit terminal index. Thus, beef cattle of high total genetic merit for a terminal index are expected to yield carcasses of superior quality and value, in comparison to their low total genetic merit counterparts, which helps ensure economic sustainability for both the primary producer and the abattoir.

### Economic Impact of Improved Terminal Total Genetic Merit

As the terminal index described in the present study is an economic selection index, cattle of higher total genetic merit are expected to contribute more profit to the producer. The ability of higher terminal index cattle to generate more carcass revenue has previously been confirmed by Connolly et al. (2016). From results in the present study, assuming a 120-d finishing period, cattle in the very high terminal index stratum are expected to eat 1,087 MJ (9.06 MJ difference in REI between extreme strata × 120 d) less than cattle in the very high index stratum for the same growth rate and live-weight; assuming a feed cost of €0.025/MJ, this represents a feed cost saving of €2,686 for a producer finishing 100 animals. Moreover, the economic benefit of the better feed and production efficiency of higher terminal index cattle can be further quantified in the current study by using the economic weights used in the terminal index and the observed phenotypic differences in DM intake, carcass weight, carcass conformation, and carcass fat between terminal index strata described herein. Therefore, relative to cattle in the very low terminal index stratum, cattle in the very high terminal index stratum are expected to generate €139.62 more profit per animal [i.e., (−0.63 kg DM/d × −€38.63) + (25.02 kg × €3.14) + (1.82 units × €14.77) + (−1.24 units × −€7.86)]. Based on the differences between extreme terminal index strata in the EBVs for daily feed intake, carcass weight, carcass conformation, and carcass fat, cattle in the very high terminal index stratum were expected to be €151.84 more profitable in comparison to cattle in the very low terminal index stratum. Therefore, the greater expected profit based on the observed phenotypic differences between strata in the present study is comparable to what would be expected based on their respective difference in EBVs. The terminal index described herein, however, also includes calving performance, perinatal mortality, and docility which, alongside traits which are not included in the terminal index, such as age at slaughter, are also expected to further contribute to the profitability of the beef production system. Genetic gain in the Irish terminal index between the years 2005 and 2018 has been approximately €35 based on an EBV scale (Twomey et al., 2020); this translates to an increase in profit for the beef industry of €35 million per one million animals slaughtered.

## CONCLUSIONS

The potential improvements in animal performance presented in the current study from selection on a holistic breeding index are clear for both feed and production efficiency. Multiple phenotypic measures of efficiency can be improved in a cattle population where selection takes place using such an all-encompassing total merit selection index which includes feed intake and the energy sinks of carcass weight, carcass conformation, and carcass fat among others. Not only are heavier carcasses of superior quality and value being achieved in genetically elite animals, these animals also have a greater proportion of carcass weight relative to live-weight (i.e., better dressing percentage), with fewer days to slaughter and all at a lower daily feed input. The improvements in efficiency described herein still represents only feed efficiency during the finishing period prior to slaughter; in national breeding objectives, feed efficiency across the entirety of the animal’s life should also be considered. Nevertheless, the results from the present study will be useful to quantify the benefits of selection for feed and production efficiency when using a total merit index and to help instill industry confidence in the contribution of genetic selection to generation-on-generation improvements in individual animal performance and efficiency.

## Supplementary Material

txaa106_suppl_Supplementary_Tables
