# Supplementary material for: Feed and production efficiency of young crossbred beef cattle stratified on a terminal total merit index
Source: Transl Anim Sci. 2020 Jul 1;4(3):txaa106. doi: 10.1093/tas/txaa106 (PMC7381835; doi:10.1093/tas/txaa106)
Supplement: txaa106_suppl_Supplementary_Tables [file txaa106_suppl_supplementary_tables.docx]

**SUPPLEMENTARY MATERIAL**

**Supplementary Table 1.** Least squares means^1^ and pooled standard errors of the difference between least squares means (SED) for performance, efficiency, carcass, and ultrasound traits for very low, low, high and very high terminal index bulls.

| Trait^2^ | Very Low | Low | High | Very High | SED |
| --- | --- | --- | --- | --- | --- |
| *Performance* |  |  |  |  |  |
| Average daily gain, kg/d | 1.95 | 2.01 | 1.99 | 2.01 | 0.050 |
| Dry matter intake, kg/d | 14.00 | 14.27 | 13.82 | 13.35 | 0.256 |
| Metabolizable energy intake, MJ/d | 187.58^ab^ | 191.14^a^ | 184.88^ab^ | 178.51^b^ | 3.206 |
| Metabolic live-weight, kg^0.75^ | 126.4^ab^ | 130.4^a^ | 129.4^ab^ | 127.1^b^ | 1.567 |
| Pre-slaughter live-weight, kg | 714.3 | 741.0 | 735.4 | 721.0 | 11.039 |
| Age at slaughter^3^, d | 461 | 460 | 453 | 454 | 4.269 |
| Adjusted age at slaughter^4^,d | 466 | 466 | 464 | 462 | 4.240 |
| *Efficiency* |  |  |  |  |  |
| Energy conversion ratio | 100.70 | 100.74 | 97.65 | 93.80 | 2.982 |
| Relative growth rate | 0.360 | 0.356 | 0.354 | 0.368 | 0.010 |
| Kleiber ratio | 0.0158 | 0.0158 | 0.0157 | 0.0162 | 0.0004 |
| Residual energy intake, MJ/d | 2.49^a^ | 1.41^a^ | -3.67^ab^ | -8.10^b^ | 2.239 |
| REI_U_, MJ/d | 0.02^a^ | 1.34^a^ | -3.87^ab^ | -6.33^b^ | 2.560 |
| Residual gain, kg/d | -0.08^a^ | -0.06^ab^ | -0.01^ab^ | 0.07^b^ | 0.042 |
| RG_U_, kg/d | -0.10^a^ | -0.07^a^ | -0.05^ab^ | 0.03^b^ | 0.045 |
| Residual intake and gain | -0.54^a^ | -0.36^a^ | 0.31^ab^ | 1.04^b^ | 0.300 |
| RIG_U_ | -0.42^a^ | -0.44^a^ | 0.17^ab^ | 0.78^b^ | 0.370 |
| *Carcass* |  |  |  |  |  |
| Carcass weight, kg | 405.44^a^ | 426.45^ab^ | 431.05^b^ | 429.75^b^ | 6.584 |
| Carcass conformation, scale 1-15 | 11.10^a^ | 11.86^b^ | 12.24^bc^ | 12.80^c^ | 0.222 |
| Carcass fat, scale 1-15 | 7.29^a^ | 6.92^ab^ | 6.41^bc^ | 5.92^c^ | 0.234 |
| Dressing difference, kg | 308.26^ab^ | 313.86^a^ | 303.58^ab^ | 290.63^b^ | 5.504 |
| Dressing percentage, % | 57.16^a^ | 57.83^ab^ | 59.06^bc^ | 60.16^c^ | 0.375 |
| *Ultrasound* |  |  |  |  |  |
| Ultrasound fat depth, mm | 4.58^a^ | 4.16^ab^ | 3.86^ab^ | 3.51^b^ | 0.275 |
| Ultrasound muscle depth, mm | 75.22^a^ | 77.07^ab^ | 79.76^b^ | 80.90^b^ | 1.371 |
| Intramuscular fat, % | 6.04^a^ | 5.84^a^ | 5.43^ab^ | 5.15^b^ | 0.254 |

^a-d^Least squares means within a row with different subscripts differ (P < 0.05).

^1^Referrent animal was a purebred bull from a 3^rd^ parity dam slaughtered at 16 months of age.

^2^REI_U_ = residual energy intake adjusted for ultrasound fat depth; RG_U_ = residual gain adjusted for ultrasound fat depth; RIG_U_ = residual intake and gain adjusted for ultrasound fat depth.

^3^Referent animal was a purebred bull from a 3^rd^ parity dam.

^4^Referent animal was a purebred bull from a 3^rd^ parity dam slaughtered at a carcass weight of 360 kg.

**Supplementary Table 2.** Least squares phenotypic means^1^ and pooled standard errors of the difference between least squares means (SED) for performance, efficiency, carcass, and ultrasound traits for very low, low, high and very high terminal index heifers.

| Trait^2^ | Very Low | Low | High | Very High | SED |
| --- | --- | --- | --- | --- | --- |
| *Performance* |  |  |  |  |  |
| Average daily gain, kg/d | 1.22 | 1.29 | 1.19 | 1.37 | 0.067 |
| Dry matter intake, kg/d | 11.45 | 10.90 | 10.39 | 10.75 | 0.344 |
| Metabolizable energy intake, MJ/d | 138.43 | 131.91 | 125.71 | 130.13 | 4.310 |
| Metabolic live-weight, kg^0.75^ | 114.0 | 114.3 | 111.9 | 115.4 | 2.107 |
| Pre-slaughter live-weight, kg | 596.0 | 600.2 | 582.0 | 610.1 | 14.841 |
| Age at slaughter^3^, d | 523 | 517 | 515 | 512 | 5.738 |
| Adjusted age at slaughter^4^,d | 530 | 524 | 522 | 516 | 5.725 |
| *Efficiency* |  |  |  |  |  |
| Energy conversion ratio | 115.62^a^ | 105.92^ab^ | 107.46^ab^ | 97.58^b^ | 4.010 |
| Relative growth rate | 0.231 | 0.246 | 0.233 | 0.257 | 0.013 |
| Kleiber ratio | 0.0107 | 0.0114 | 0.0107 | 0.0119 | 0.0005 |
| Residual energy intake, MJ/d | 5.27^a^ | -1.91^ab^ | -4.59^ab^ | -4.89^b^ | 3.029 |
| REI_U_, MJ/d | 3.23^a^ | 1.00^ab^ | -3.04^ab^ | -5.31^b^ | 3.52 |
| Residual gain, kg/d | -0.03 | 0.03 | -0.04 | 0.10 | 0.057 |
| RG_U_, kg/d | -0.02 | 0.04 | -0.03 | 0.09 | 0.061 |
| Residual intake and gain | -0.63^a^ | 0.31^ab^ | 0.28^ab^ | 0.88^b^ | 0.406 |
| RIG_U_ | -0.42^a^ | -0.44^ab^ | 0.17^ab^ | 0.78^b^ | 0.513 |
| *Carcass* |  |  |  |  |  |
| Carcass weight, kg | 319.15 | 326.45 | 326.06 | 344.52 | 8.852 |
| Carcass conformation, scale 1-15 | 8.43^a^ | 9.11^a^ | 10.08^b^ | 10.19^b^ | 0.298 |
| Carcass fat, scale 1-15 | 8.63 | 8.24 | 7.74 | 7.66 | 0.314 |
| Dressing difference, kg | 276.24 | 273.10 | 255.26 | 265.00 | 7.399 |
| Dressing percentage, % | 53.68^a^ | 54.61^a^ | 56.28^b^ | 56.64^b^ | 0.504 |
| *Ultrasound* |  |  |  |  |  |
| Ultrasound fat depth, mm | 6.16^a^ | 5.53^ab^ | 4.87^b^ | 4.67^b^ | 0.363 |
| Ultrasound muscle depth, mm | 76.53^ab^ | 75.09^a^ | 79.41^ab^ | 81.45^b^ | 1.809 |
| Intramuscular fat, % | 6.40 | 6.79 | 6.12 | 5.94 | 0.335 |

^a-d^Least squares means within a row with different subscripts differ (P < 0.05).

^1^Referrent animal was a purebred heifer from a 3^rd^ parity dam slaughtered at 18 months of age.

^2^REI_U_ = residual energy intake adjusted for ultrasound fat depth; RG_U_ = residual gain adjusted for ultrasound fat depth; RIG_U_ = residual intake and gain adjusted for ultrasound fat depth.

^2^Referent animal was a purebred heifer from a 3^rd^ parity dam.

^3^Referent animal was a purebred heifer from a 3^rd^ parity dam slaughtered at a carcass weight of 360 kg.

**Supplementary Table 3.** Least squares phenotypic means^1^ and pooled standard errors of the difference between least squares means (SED) for performance, efficiency, carcass, and ultrasound traits for very low, low, high, and very high terminal index steers.

| Trait^2^ | Very Low | Low | High | Very High | SED |
| --- | --- | --- | --- | --- | --- |
| *Performance* |  |  |  |  |  |
| Average daily gain, kg/d | 1.52 | 1.46 | 1.49 | 1.51 | 0.050 |
| Dry matter intake, kg/d | 12.33 | 12.14 | 11.88 | 11.78 | 0.256 |
| Metabolizable energy intake, MJ/d | 148.90 | 146.62 | 143.48 | 142.36 | 3.205 |
| Metabolic live-weight, kg^0.75^ | 119.7 | 121.0 | 118.5 | 120.5 | 1.567 |
| Pre-slaughter live-weight, kg | 644.2 | 649.9 | 632.4 | 647.5 | 11.032 |
| Age at slaughter^3^, d | 579^a^ | 573^ab^ | 584^ab^ | 560^b^ | 4.225 |
| Adjusted age at slaughter^4^,d | 598^a^ | 592^ab^ | 590^b^ | 585^b^ | 4.190 |
| *Efficiency* |  |  |  |  |  |
| Energy conversion ratio | 101.77 | 105.10 | 99.30 | 97.59 | 2.982 |
| Relative growth rate | 0.278 | 0.264 | 0.274 | 0.271 | 0.010 |
| Kleiber ratio | 0.0129 | 0.0123 | 0.0127 | 0.0126 | 0.0004 |
| Residual energy intake, MJ/d | 2.50 | 0.40 | 0.18 | -3.94 | 2.220 |
| REI_U_, MJ/d | 0.57 | -2.17 | 2.63 | -2.26 | 3.011 |
| Residual gain, kg/d | 0.04 | 0.00 | 0.05 | 0.06 | 0.041 |
| RG_U_, kg/d | 0.06 | 0.00 | 0.03 | 0.03 | 0.053 |
| Residual intake and gain | -0.06 | -0.04 | 0.17 | 0.63 | 0.297 |
| RIG_U_ | 0.21 | 0.21 | -0.15 | 0.36 | 0.435 |
| *Carcass* |  |  |  |  |  |
| Carcass weight, kg | 347.04^a^ | 356.50^ab^ | 354.62^ab^ | 372.43^b^ | 6.581 |
| Carcass conformation, scale 1-15 | 8.48^a^ | 9.19^ab^ | 9.58^b^ | 10.48^c^ | 0.221 |
| Carcass fat, scale 1-15 | 7.57^a^ | 7.16^ab^ | 6.73^bc^ | 6.19^c^ | 0.233 |
| Dressing difference, kg | 297.17^a^ | 293.36^ab^ | 277.50^bc^ | 275.14^c^ | 5.495 |
| Dressing percentage, % | 53.84^a^ | 54.82^a^ | 56.09^b^ | 57.56^c^ | 0.373 |
| *Ultrasound* |  |  |  |  |  |
| Ultrasound fat depth, mm | 5.60^a^ | 5.07^a^ | 3.96^b^ | 3.41^b^ | 0.319 |
| Ultrasound muscle depth, mm | 73.86^a^ | 77.69^ab^ | 78.02^ab^ | 79.65^b^ | 1.587 |
| Intramuscular fat, % | 6.35^a^ | 5.53^ab^ | 5.38^b^ | 5.15^b^ | 0.294 |

^a-d^Least squares means within a row with different subscripts differ (P < 0.05).

^1^Referrent animal was a steer from a purebred steer from a 3^rd^ parity dam slaughtered at 20 months of age.

^2^REI_U_ = residual energy intake adjusted for ultrasound fat depth; RG_U_ = residual gain adjusted for ultrasound fat depth; RIG_U_ = residual intake and gain adjusted for ultrasound fat depth.

^3^Referent animal was a purebred steer from a 3^rd^ parity dam.

^4^Referent animal was a purebred steer from a 3^rd^ parity dam slaughtered at a carcass weight of 360 kg.
